# Study protocol, randomized controlled trial: reducing symptom burden in patients with heart failure with preserved ejection fraction using ubiquinol and/or D-ribose

**DOI:** 10.1186/s12872-018-0796-2

**Published:** 2018-04-02

**Authors:** Janet D. Pierce, Diane E. Mahoney, John B. Hiebert, Amanda R. Thimmesch, Francisco J. Diaz, Carol Smith, Qiuhua Shen, Dinesh Pal Mudaranthakam, Richard L. Clancy

**Affiliations:** 1 School of Nursing, University of Kansas, 3901 Rainbow Blvd, Kansas City, KS 66160 USA; 2 Department of Biostatistics, University of Kansas Medical Center, 3901 Rainbow Blvd, Kansas City, KS 66160 USA; 3 Department of Molecular and Integrative Physiology, University of Kansas Medical Center, 3901 Rainbow Blvd, Kansas City, KS 66160 USA

**Keywords:** Heart failure with preserved ejection fraction (HFpEF); ubiquinol, D-ribose, Bioenergetics

## Abstract

**Background:**

Heart failure (HF), the leading cause of morbidity and mortality in the US, affects 6.6 million adults with an estimated additional 3 million people by 2030. More than 50% of HF patients have heart failure with preserved left ventricular ejection fraction (HFpEF). These patients have impaired cardiac muscle relaxation and diastolic filling, which investigators have associated with cellular energetic impairment. Patients with HFpEF experience symptoms of: (1) fatigue; (2) shortness of breath; and (3) swelling (edema) of the lower extremities. However, current HF guidelines offer no effective treatment to address these underlying pathophysiologic mechanisms. Thus, we propose a biobehavioral symptom science study using ubiquinol and D-ribose (therapeutic interventions) to target mitochondrial bioenergetics to reduce the complex symptoms experienced by patients with HFpEF.

**Methods:**

Using a randomized, double-blind, placebo-controlled design, the overall objective is to determine if administering ubiquinol and/or D-ribose to HFpEF patients for 12 weeks would decrease the severity of their complex symptoms and improve their cardiac function. The measures used to assess patients’ perceptions of their health status and level of vigor (energy) will be the Kansas City Cardiomyopathy Questionnaire (KCCQ) and Vigor subscale of the Profile of Mood States. The 6-min walk test will be used to test exercise tolerance. Left ventricular diastolic function will be assessed using innovative advanced echocardiography software called speckle tracking. We will measure B-type natriuretic peptides (secreted from ventricles in HF) and lactate/ATP ratio (measure of cellular energetics).

**Discussions:**

Ubiquinol (active form of Coenzyme Q10) and D-ribose are two potential treatments that can positively affect cellular energetic impairment, the major underlying mechanism of HFpEF. Ubiquinol, the reduced form of CoQ10, is more effective in adults over the age of 50. In patients with HFpEF, mitochondrial deficiency of ubiquinol results in decreased adenosine triphosphate (ATP) synthesis and reduced scavenging of reactive oxygen species. D-ribose is a substrate required for ATP synthesis and when administered has been shown to improve impaired myocardial bioenergetics. Therefore, if the biological underpinning of deficient mitochondrial ATP in HFpEF is not addressed, patients will suffer major symptoms including lack of energy, fatigue, exertional dyspnea, and exercise intolerance.

**Trial registration:**

ClinicalTrials.gov Identifier: NCT03133793; Data of Registration: April 28, 2017.

## Background

The public burden of heart failure (HF) is manifested by its staggering cost ($39.2 billion in healthcare-related costs/year) in the US [1], while the personal burden of HF includes debilitating symptoms [2]. It is estimated that there is a 20% lifetime risk of developing HF at age 40 and that 870,000 new cases will be diagnosed yearly in adults who are 55 years of age or older [3]. In 2014, the Food and Drug Administration reported, “the burden of HFpEF was considerable and is projected to worsen” and “there are no approved therapies available for reducing symptoms” [4]. Beginning January 2012, the Centers for Medicare and Medicaid Services began reducing payments to hospitals for HF patients readmitted within 30 days of hospital discharge. Of the patients hospitalized with HF, more than 50% have diastolic HF, which is termed heart failure with preserved ejection fraction (HFpEF) [5]. These patients have a left ventricular ejection fraction (LVEF) of more than 45 to 50% and symptoms that include lack of energy (fatigue), shortness of breath, peripheral edema, and reduced quality of life [6, 7]. The most recent American College of Cardiology (ACC)/American Heart Association (AHA) HF guidelines for HFpEF acknowledge “huge gaps” in understanding effective treatments for patients with HFpEF [8]. This study will provide an opportunity to evaluate the comparative effectiveness of alternative approaches for reducing symptom burden in patients with HFpEF.

This study of ubiquinol (active form of Coenzyme Q10) and D-ribose in patients with HFpEF will significantly improve scientific understanding of myocardial bioenergetics. Ubiquinol and D-ribose are naturally produced in the body [9–11]. One of the main functions of these products is to promote cardiac energy metabolism by increasing mitochondrial adenosine triphosphate (ATP) production [12]. Ubiquinol and D-ribose exert this physiological benefit of enhancing cardiac function via two different bioenergetic pathways [13–15]. As an efficient electron carrier, ubiquinol plays an important role in transporting electrons in the electron transport chain (ETC), which produces the proton gradient needed for phosphorylation of ADP and creating ATP. In contrast, D-ribose provides a source for synthesis of adenosine monophosphate (AMP) to ensure there is sufficient ADP to form ATP [16–18]. Therefore, administering these supplements should augment ATP synthesis, increasing both cellular and behavioral energy. The potential benefits of administering ubiquinol and/or D-ribose to patients with HFpEF are increased myocardial energy and diastolic relaxation, which would significantly reduce their cardiac symptoms. We plan to measure HFpEF symptoms using the Kansas City Cardiomyopathy Questionnaire (KCCQ), Vigor scale, and 6-min walk test (6MWT). Biological measures will include myocardial wall motion, lactate/ATP ratio, and B-type natriuretic peptides (BNP).

Novel discoveries in biobehavioral symptom science require integration of information from the patient’s biologic processes, physiologic pathways, and behaviors to treat and manage symptoms of diseases such as HFpEF [19, 20]. We plan to use biobehavioral symptom science to identify complex symptoms that can be characterized with biological and clinical data. In this study, symptoms of HFpEF patients that result in a person’s feeling a lack of energy will be measured using a questionnaire to evaluate the patient’s level of vigor and overall health status. We will also measure their lactate/ATP ratio, cardiac performance, BNP, and conduct a 6MWT. This study could assist in illuminating a therapeutic clinical intervention (ubiquinol and/or D-ribose) for patients with HFpEF by quantifying subjective symptom experiences and measuring biologic and physiologic underpinnings of the symptoms.

We plan to use a sophisticated echocardiographic software program called speckle tracking to measure myocardial wall tension and motion to evaluate diastolic function. In addition, we will quantify our term “energy” using point-of-contact instrumentation to measure lactate/ATP ratio and BNP concentration. The two important outcomes for patients with HFpEF are improved heart function and patient energy. If a patient with HFpEF takes ubiquinol and/or D-ribose, we hypothesize that the increased cellular bioenergetics would decrease myocardial stiffness, resulting in improved cardiac output and level of vigor. With more energy, the patient should have improved health status in the five KCCQ domains (i.e., clinical summary, functional status, self-efficacy, social interactions, and quality of life). Thus, if the aims are achieved, patients with HFpEF will have successful symptom management and improved assessment of diastolic function. In addition, we will determine whether the comparative effectiveness of the treatments will maintain or increase ATP levels, prevent additional stiffness of the heart, and thus improve cardiac function, thereby reducing symptoms. A reduced myocardial tissue content of CoQ10 has been demonstrated in patients with HF, correlating with the severity of symptoms and the degree of left ventricular dysfunction [21]. According to Sharma (2016), the pathophysiological rationale for the use of CoQ10 in HFpEF exists, and well-conducted clinical trials will be required to demonstrate how CoQ10 decreases the signs and symptoms in this population [22]. This study will produce new knowledge about comparative benefits and outcomes that is significant to patients with HFpEF and that will help these patients make informed critical decisions about using ubiquinol and/or D-ribose.

Since HFpEF is related to changes in cellular bioenergetics, it is important to evaluate behavioral measures of energy such as the Vigor scale as well as biological clinical outcomes. After meeting with 30 HFpEF patients and 10 caregivers during a patient-centered focus group, we found they were extremely interested in the question of whether ubiquinol, D-ribose, or both could improve their health status (i.e., reduce their shortness of breath, increase their ability to perform daily activities, etc.) and their energy levels. With the advanced speckle tracking echocardiogram images, lactate/ATP ratio, and BNP measurements, we can answer that question. Our data will help us compare the effectiveness of ubiquinol and D-ribose with usual treatment in this patient population.

### Aim and hypotheses

The overall objective is to determine if administering ubiquinol and/or D-ribose to HFpEF patients for 12 weeks will decrease the severity of their complex symptoms and improve their cardiac function. For this study, there are two aims and six hypotheses that are listed in Table 1.

**Table 1:** List of aims and hypothesis for the study

AIM 1: To determine the effects of oral ubiquinol, D-ribose, or a combination of the two administered over 12 weeks on symptoms accompanying low bioenergetics in patients with HFpEF.
Hypothesis #1: Ubiquinol (600 mg daily), D-ribose (15 g daily), or a combination of the two will enhance the health status of patients with HFpEF as measured by the Kansas City Cardiomyopathy Questionnaire.
Hypothesis #2: Ubiquinol (600 mg daily), D-ribose (15 g daily), or a combination of the two will increase the level of vigor in patients with HFpEF as measured by the Vigor subscale of the Profile of Mood States.
AIM 2: To determine the effects of oral ubiquinol, D-ribose, or a combination of the two over 12 weeks on biological measures in patients with HFpEF.
Hypothesis #3: Ubiquinol (600 mg daily), D-ribose (15 g daily), or a combination of the two will improve left ventricular diastolic function measured by advanced echocardiographic imaging in patients with HFpEF.
Hypothesis #4: Ubiquinol (600 mg daily), D-ribose (15 g daily), or a combination of the two will increase the distance that patients with HFpEF can walk in 6 min.
Hypothesis #5: Ubiquinol (600 mg daily), D-ribose (15 g daily), or a combination of the two will decrease venous blood B-type natriuretic peptide (BNP) levels in patients with HFpEF.
Hypothesis #6: Ubiquinol (600 mg daily), D-ribose (15 g daily), or a combination of the two will decrease the lactate/ATP ratio in patients with HFpEF.

## Methods/design

**Fig. 1:**
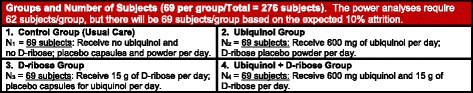
Study groups and Number of Subjects per Group

This study is a randomized, double-blinded, controlled trial, with an aim to enroll a total of 276 subjects (69 subjects in each arm) (Fig. 1). The participants will receive usual care for HFpEF. Subjects will be required to speak English and the informed consent and patient information will be written at the 8th grade level. We will collaborate with our Human Subject Protection Program to ensure our consent form complies with the US Department of Health and Human Services federal guidelines and obtain informed consent from eligible patients.

To ensure adequate enrollment, the University of Kansas Medical Center database called Healthcare Enterprise Repository for Ontological Narration will be used. It provides clinical/translational investigators with the numbers of patients at Kansas University Hospital who meet the inclusion criteria for clinical trials and contact information for patients who sign up with the University of Kansas Medical Center’s Frontiers participant registry. Based on this database, there will be approximately 7100 subjects with HF available to be screened for enrollment in this study. In addition to this, the Pioneers recruitment registry, a community-based participant registry will also be used to facilitate recruitment.

### Study population and environment

The Institutional Review Board (IRB) will first approve the research protocol, and all subjects will be provided with a written informed consent. After consent, the subjects will be given visual and written directions to the Clinical and Translational Science Unit (CTSU) contact phone and address information. Eligible participants who are > 50 years of age, male and female with HFpEF, will be recruited from the chronic heart failure clinic at a University hospital, Healthcare Enterprise Repository for Ontological Narration database, or the Pioneers Recruitment Registry. We will oversample minority populations to increase the generalizability of data for minority groups (30% Black/African Americans (AA), 30% Hispanic/Latino, 30% White, and 10% American Indian/Alaska Native, Asian, Native Hawaiian/Pacific Islander). All data will be gathered for the explicit purposes of this study using procedures to ensure confidentiality. All personal data will be identified by code number only. A list that links the assigned code number to the subject’s name will be kept separately in a locked file located in the School of Nursing. Only the Project Director and Research Associate will have the access to individually identifiable private information about study subjects.

### Data collection

In order to ensure quality and consistency in the data collection required by the study protocol, numerical response categories in data elements will be utilized to allow summarization and analysis. In addition, redundant data elements and unnecessary data will be avoided. The Comprehensive Research Information System called CRIS powered by Velos eResearch is a web-based clinical information management system that will be used for the clinical data collection and managing database. It is compliant with the National Institutes of Health (NIH) reporting standards (e.g., role-based access, password-protected files, storage on secure servers, e-signature). In addition to housing trial data, current versions of the protocol, consent and other IRB-approved documents are available on CRIS.

### Eligibility and recruitment

To participate in the study, the patients are required to meet all of the inclusion and exclusion criteria (Table 2). If all criteria are met, the patient will be invited by study personnel to participate in the study and informed consent will be obtained. Once the patients have consented, they will be provided a 1-week supply of placebo ubiquinol pills (instructed to take 2 pills/day) and placebo D-ribose powder (instructed to take 3 scoops/day) as a pre-randomization run-in period to evaluate adherence to the supplements. Subjects will be scheduled for an appointment at the CTSU, located in the United States, where all data will be collected 1 week following the run-in period. Subjects that demonstrate an 80% compliance rate of ubiquinol intake (as evidenced by pill count) and an 80% adherence rate of D-ribose powder intake (as evidenced by weight of the containers) will be eligible to be randomized into a group assignment.

**Table 2:** Inclusion and exclusion criteria for the study

Inclusion Criteria
1. Age 50 years old or older
2. Left ventricular ejection fraction (EF) ≥ 50% documented by an echocardiogram within 12 months of enrollment
3. Diagnosis of HFpEF within a 12-month period
4. Symptoms of dyspnea and New York Heart Association (NYHA) Classification II-or greater symptoms, and a diagnosis of HFpEF defined by ***ONE*** of the following:
4a. Previous hospitalization for HF with radiographic evidence (pulmonary venous hypertension, vascular congestion, interstitial edema, or pleural effusion) of pulmonary congestion
4b. Invasive hemodynamics
4c. Echocardiographic evidence of diastolic dysfunction
4d. Elevated NT-proBNP (> 400 pg/mL) or BNP (> 200 pg/mL)
4e. No use of intermittent sublingual nitroglycerin within 12 months
5. Willingness to provide informed consent
6. Have a telephone or reliable phone contact
7. Have their own means of transportation to the study site
Exclusion Criteria
1. Acute coronary syndrome in the past 12 weeks
2. Severe valvular heart disease
3. Severe cardiac fibrosis (galectin-3 level > 26 ng/ml)
4. Constrictive pericardium
5. Pulmonary fibrosis
6. Congenital heart disease
7. Hypertrophic or infiltrative cardiomyopathy
8. Heart transplant
9. Left ventricular assist device
10. HF associated hospital admission or ED visit within past 30 days
11. Percutaneous coronary intervention in the last month
12. Renal (Creatinine > 3) and/or severe hepatic dysfunction
13. Severe cognitive impairment
14. No current consumption of any CoQ10 (ubiquinol) or D-ribose supplements

Recruited subjects will be assigned to one of the four groups using a list created by a computer-based random number generator. All supplements and the placebo will be indistinguishable in packaging and will be distributed by the Project Director or Research Associate independent of the Principal Investigator (PI) so that the allocation of subjects to a treatment or placebo group will be concealed from both subjects and other research personnel. Strategies to optimize retention and improve adherence is to include follow-up phone calls at 3, 6, and 9 weeks into the study and subject incentives in the amount of $50 at the end of each visit and another $50 at completion of the study. A list of any outcome data to be collected for participants who discontinue or deviate from intervention protocols is stated on Table 3.

**Table 3:** Protocol deviations

DEVIATION CATEGORIES: A. Safety B. Informed Consent C. Eligibility D. Protocol implementation E. Other, specify in log DEVIATION CODES: Numbers listed by the sample protocol deviations	Eligibility (Category C) 12. Participant did not meet eligibility criterion 13. Randomization of an ineligible participant 14. Participant randomized prior to completing Baseline Assessment, etc. 15. Randomization and/or treatment of participant prior to IRB approval of protocol 16. Other, specify in log
Safety (Category A) 1. Not reporting an SAE within 24 h 2. Laboratory tests not done 3. AE/SAE is not reported to IRB 4. Other, specify in log	Protocol implementation (Category D) 17. Failure to keep IRB approval up to date 18. Participant receives wrong treatment 19. Participant seen outside visit window 20. Use of unallowable concomitant treatments 21. Prescribed dosing outside protocol guidelines 22. Missed assessment 23. Missed visit 24. Other, specify in log
Informed Consent (Category B) 5. Failure to obtain informed consent 6. Consent form used was not current IRB-approved version 7. Consent form does not include updates or information required by IRB 8. Consent form missing 9. Consent form not signed and dated by participant 10. Consent form does not contain all required signatures 11. Other, specify in log	

### Study intervention

A local pharmacy will prepare, package, and label containers for study placebo ubiquinol capsules and D-ribose powder. This pharmacy will provide labels and empty containers for the study treatment, ubiquinol and D-ribose. The Project Director and Research Associate will prepare and package the ubiquinol capsules and D-ribose powder. This entails repacking supplements obtained from the original distributers and replacing them into the empty containers obtained from the pharmacy. All supplements and placebo packaging will be indistinguishable in appearance. Microcrystalline cellulose will be used as the placebo substance for ubiquinol and D-ribose.

Each label will include study identification number, medication of name written as “D-Ribose 5 g or Placebo,” and “Ubiquinol 300 mg or Placebo,” directions for use, lot number, current date, and an expiration date. Only the Research Statisticians, Project Director, and Research Associate will have access to the participant assignment list and respective identification number.

Control: Subjects will receive no ubiquinol and no D-ribose; they will receive 2 placebo capsules and 3 scoops of placebo powder per day for 12 weeks. A total of 175 placebo capsules (2/day) and 270 scoops of placebo powder will be distributed.

Ubiquinol group: Subjects will receive 600 mg (300 mg capsules × 2) of ubiquinol/day and 3 scoops of placebo powder per day for 12 weeks. A total of 175 ubiquinol capsules and 270 scoops of placebo powder will be distributed.

D-ribose group: Subjects will receive 2 placebo capsules/day and 15 g of D-ribose/day (3 scoops) for 12 weeks. A total of 175 placebo capsules (2/day) and 174,000 g of D-ribose (15 g/day) will be distributed.

Ubiquinol + D-ribose group: Subjects receive 600 mg (300 mg capsules × 2) ubiquinol/day and 15 g of D-ribose/day (3 scoops) per day for 12 weeks. A total of 175 ubiquinol capsules and 174,000 g of D-ribose (15 g/day) will be distributed.

At the end of the baseline visit, the appropriate investigational supplements will be provided to the participant based on group assignment. The respective identification number linking the investigative product to the participant name will be documented on a designated form and stored in a locked cabinet. Only the Project Manager and Research Associate will have access to this locked cabinet.

Investigational supplements will be stored at CTSU in a temperature controlled medication storage room that is monitored by a thermometer set at 20.83 °C/69.5 °F. Once a day during hours of operation, CTSU staff manually document room temperature on a paper log, which is located in the medication storage room. Drugs will be wasted if the medication storage room temperature falls outside of the parameters specified by drug manufacturer storage requirements.

Ubiquinol will be purchased from the manufacturer in 300 mg capsules distributed in qualities of 60 per bottle. Participants will be instructed to take two capsules daily. The D-ribose powder will be purchased from the manufacturer in bulk supply. Any unused supplements that are returned by study participants will be disposed of through the Kansas University Medical Center Safety Office and documented.

### Randomization

Random assignment in a 1:1:1:1 ratio to one of four groups will be performed. Randomization minimizes differences between groups by equally distributing participants with particular characteristics among four arms. We will target about 69 subjects per group in total, including 21 Black/African Americans in each group.

To randomize a new participant, demographic data and confirmation of eligibility must first be entered into CRIS. To ensure a balance design with respect to race, two randomization lists will be used, one for Black/African Americans, the other for other races. Once screened, CRIS automatically assigns the next available trial ID and identifies the next treatment arm assignment from the randomization table, based on race. Once randomized to an assigned group, each participant will be assigned a respective study identification or code number that links to participant name and assignment. This randomization assignment will be documented on a designated participant identification list.

### Blinding

All parties, with the exception of the Project Manager, Statisticians, and Research Associate will be blinded to the treatment groups and will not know if the participants are receiving the experimental or control intervention. Should unblinding occur the following would be recorded: The identification of the unblinded participant, the reason for unblinding, the study staff person responsible for unblinding, and a list of person(s) who have been unblinded.

**Fig. 2:**
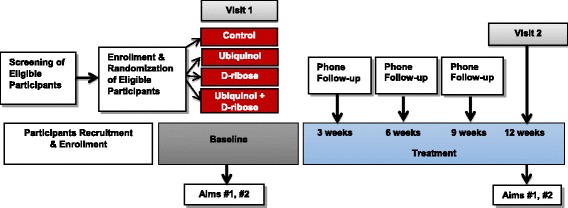
Study design and groups

All subjects will complete the demographic form at baseline. The KCCQ and Vigor scale from the Profile of Mood States (POMS) will be completed in CTSU visits at baseline and 12 weeks. The patient will be escorted to a private room and asked to remove clothing above the waist. A disposable gown will be provided and the patient will lay supine on the bed while an echocardiogram is completed. Approximately 0.5 ml of blood will be collected via finger stick to measure lactate, ATP, and BNP. Next, the subject will complete a 6MWT including the Borg scale. Approximately 2 h will be required for each subject’s visit to the CTSU. Follow-up calls will occur at 3, 6, and 9 weeks during the trial (Fig. 2).

### Kansas City cardiomyopathy questionnaire (KCCQ)

We will assess patients’ perceptions of their symptoms using the KCCQ, a self-administered questionnaire that measures patients’ perceptions of five domains of their health status relevant to HFpEF [23]. These domains are: (1) physical limitations, (2) symptoms, (3) self-efficacy, (4) quality of life, and (5) social interference. The KCCQ consists of 24 items to which patients respond on a scale indicating limitations due to HF. For example, for activities such as dressing, doing yard work, or climbing a flight of stairs, patients will be asked to check a response that indicates the degree to which HF has limited their ability: “Extremely Limited,” “Quite a bit Limited,” “Moderately Limited,” “Slightly Limited,” “Not at all Limited,” or “Limited for other reasons or did not do the activity.” Another item is: “Over the past 2 weeks, how many times has shortness of breath limited your ability to do what you wanted?” Response alternatives are: “All of the time,” “Several times,” “At least once a day,” “3 or more times per week but not every day,” “1-2 times per week,” “Less than once a week,” and “Never over the past 2 weeks.” The KCCQ requires approximately 4 to 6 min to complete and has been used in more than a hundred studies. It has excellent psychometric properties and clinical usefulness. We will use the KCCQ to compare the changes in health status of patients with HFpEF taking ubiquinol, D-ribose, ubiquinol + D-ribose, or placebo.

### Vigor subscale

Using the Vigor subscale from POMS questionnaire [24–26], patients will rate themselves on eight adjectives (lively, active, energetic, cheerful, alert, full of pep, carefree, and vigorous) on a five-point scale (0 = not at all, 1 = a little, 2 = moderately, 3 = quite a bit, and 4 = extremely). The Vigor scale has very high internal consistency (Cronbach’s alpha = 0.90), and it takes only 1 min to complete. In many studies, it has been found to be effective for assessing vigor changes associated with exercise [27–29]. The KCCQ and POMS Vigor scale will be administered to patients after they have been sitting quietly for 10 min at the beginning of each visit (baseline and 12 weeks). The total time for patients to complete both questionnaires will be 5 to10 minutes. We anticipate that during the course of treatment the patients taking these supplements will report increasingly greater improvements in health status, daily functioning, and levels of energy.

### Echocardiographic measurements

Two-dimensional Doppler echocardiography is a safe imaging technique frequently used to assess HFpEF. However, LV diastolic dysfunction is difficult to assess using standard echocardiography [30]. Speckle tracking echocardiography is a novel, objective, and non-invasive advanced imaging technology that provides an accurate and precise way to assess diastolic ventricular function [31, 32]. It allows investigators to easily calculate and assess myocardial deformation (rotation, torsion, and strain) and tissue velocity, which are important parameters for left ventricular diastolic function [33]. In this study, a speckle tracking echocardiography technician will perform the echocardiograms during each visit (baseline and 12 weeks). We will determine ejection fraction (EF) and the ratio of mitral peak velocity of early filling (E) to early diastolic mitral annular velocity (e’) (E/e’ ratio) (stroke volume, end diastolic volume, EF) from the echocardiogram. Using speckle-tracking software, we will measure myocardial deformation, rotation, torsion, strain, strain rate, and tissue velocity. Changes in the early apical diastolic untwisting rate (rotR) and negativity of time from apical peak diastolic untwist (trotR) to mitral valve opening (MVO) are two major variables that reflect diastolic function. Decreases in rotR and trotR are indications of diastolic dysfunction [34]. We anticipate that ubiquinol and/or D-ribose will change rotR, trotR, strain, strain rate, and tissue velocity.

### 6 min walk test (6MWT)

Exercise-based testing is essential in evaluating the functional capacity and response to therapy and prognosis of patients with HF [35]. The 6MWT is a simple clinical tool which has been shown to be linearly related to oxygen uptake [36]. We will examine the effects of ubiquinol and/or D-ribose on the 6MWT at baseline and 12 weeks. Participants will be asked to wear appropriate clothes and shoes and walk the longest distance possible in 6 min. Prior to exercising, there will be a 10-min rest period during which heart rate and blood pressure will be recorded. Subjects will walk indoors on a flat, straight 30-m path marked every 3 m, and they will be informed that they can slow down or stop at any time. A one-lap demonstration will be completed before the test begins. The distance walked will be recorded. Following the 6MWT, heart rate and blood pressure will be recorded. Lactate concentration will be measured with a portable i-STAT handheld instrument using a CG4+ cartridge that requires 100 μL of blood and takes 2 min to complete. Lactate provides a measure of the oxygen debt occurring during exercise and correlates directly with reported dyspnea and fatigue. The patient will also record perceived dyspnea and fatigue at baseline and at the end of each walk by using the Borg scale, where 0 = nothing at all, 5 = severe, and 10 = very severe. Words of encouragement will be offered during the walk and water provided afterward.

### B-type natriuretic peptides (BNP)

B-type natriuretic peptides are a cardiac neurohormone secreted from the atria and ventricles in response to volume expansion [37]. Serum levels of BNP increase in cardiac disease conditions associated with increased atrial and ventricular stretch and left ventricular diastolic filling pressures. BNP concentration will be measured using a portable i-STAT handheld analyzer. A blood sample (100 μl) will be obtained from a finger stick after the subjects have completed the questionnaires at baseline and 12 weeks. We will measure BNP concentration at each visit. Prior to running the BNP test, the cartridge initiates a series of preset quality control diagnostics, from monitoring the quality of the sample to validating the reagent.

### Adenosine triphosphate (ATP)

CoQ10 is a key component in mitochondrial bioenergy transfer [14, 38]. Its enzymatic processes facilitate electron transfer in the generation of ATP. CoQ10 functions in ETC from enzyme complex I and enzyme complex II to complex III [39]. Thus, CoQ10 is critical in ATP generation because 95% of ATP generation is via ETC [40]. D-ribose is a naturally occurring compound and a simple sugar molecule that has been shown to increase cellular energy synthesis in the heart. The myocardium’s ability to resynthesize ATP is then limited by the supply of D-ribose [41, 42], and supplementing D-ribose would increase ATP production.

We plan to measure the effects of ubiquinol and/or D-ribose on mitochondrial ATP production by using an indirect measure (serum lactate/ATP ratio). A capillary finger stick blood sample will be obtained after subjects have completed the questionnaires and echocardiograms. We will use 5 μL of blood for the ATP measurements and place it in distilled water. Using a probe called the AquaSnap Total system, ATP in solution will be measured.

### Statistical analyses

Our power computations described suggest including 62 subjects in each of the four study groups (ubiquinol, D-ribose, ubiquinol + D-ribose, and placebo). Since we expect about a 10% attrition rate, we will include 69 subjects in each group for a total of 276 subjects who will be randomized to one of the four groups. Efforts will be made to recruit 50% females. We will oversample minority populations to increase the generalizability of data for minority groups (30% Black/AA, 30% Hispanic/Latino, 30% White, and 10% Other). In each group there will be Black/AA = 21, Hispanic/Latino = 21, White = 21, American Indian/Alaska Native, Asian, Native Hawaiian/Pacific Islander = 6 (Total = 69).

For hypotheses #1 and #2**,** the change in the KCCQ Clinical Summary score from baseline to the end of the 12 weeks of treatment will be computed for each patient as KCCQ score at 12 weeks minus KCCQ score at baseline. A two-tailed independent samples t-test will test the null hypothesis of no difference in mean change between patients on ubiquinol and patients on placebo. Similar tests will be used to compare D-ribose with placebo, ubiquinol + D-ribose with placebo, and ubiquinol versus ubiquinol + D-ribose. Our pilot study with ten subjects also found that, after 12 weeks of treatment, all subjects with ubiquinol or ubiquinol + D-ribose had an increase in the KCCQ Clinical Summary score of at least 10 points. The combined estimated standard deviation for the increase was 12.3 points. This implies that Cohen effect sizes of at least 0.81 (= 10/12.3) are clinically important. Therefore, a sample size of 62 subjects per group will provide at least 98% power to detect this or larger effect sizes at a nominal significance level of 0.05 (with Bonferroni correction for four comparisons). Analogous two-tailed independent samples *t*-tests will be used to compare changes in POMS scores. According to a linear regression of changes in POMS Vigor scores versus changes in the KCCQ Clinical Summary scores using our pilot data, a 10-point increase in KCCQ Clinical Summary scores predicted about a 6.5 point increase in Vigor scores. Thus, we believe that a 6.5-point increase in vigor in our subjects will be of clinical importance. Our pilot study also yielded an estimated standard deviation of 3.6 for 12 week changes in Vigor score, which implies that Cohen effect sizes of at least 1.8 are clinically important. Our sample sizes will provide 99% power to detect this or larger effect sizes at a nominal 0.05 significance level with Bonferroni correction for four comparisons. If assumptions of the t-test are not satisfied, transformations or the Mann-Whitney test will be applied. The five subjects of our pilot study who received ubiquinol showed a significant increase of 16.3 points in KCCQ Clinical Summary scores with respect to baseline (*t* = 5.23, df = 4, two-tailed paired samples *p* = 0.0064). Similarly, the other five subjects of our pilot study who received ubiquinol + D-ribose showed a significant increase of 30 points with respect to baseline (*t* = 5.10, df = 4, two-tailed paired samples *p* = 0.007). The 13.7-point increase produced by the independent effect of D-ribose was borderline significant (*t* = 2.06, df = 9, two-tailed independent samples *p* = 0.08). These results from our small pilot study suggest a high likelihood that ubiquinol, and probably D-ribose, will produce clinically important increases of 10 points in KCCQ scores, which gives additional confidence in the success of our proposed study and in the potential clinical applications of ubiquinol.

For hypothesis #3, the change in the E/e’ ratio from baseline to the end of 12 weeks of treatment will be computed for each patient as E/e’ ratio at 12 weeks minus E/e’ ratio at baseline. A two-tailed independent samples t-test will be used to test the null hypothesis of no difference in mean E/e’ ratio change between patients on ubiquinol and patients on placebo. Analogous computations and tests will be made to examine the effects of ubiquinol, D-ribose, and the combination of ubiquinol with D-ribose on PAP changes. Our pilot data showed a substantially strong and clinically important Cohen effect size of ubiquinol on E/e’ ratio (− 3.5). With 62 subjects per group, our t-test will have a very large power of 99% to detect this or larger effect sizes with a 0.05 level of significance (with Bonferroni correction for 4 comparisons, since both D-ribose alone and combined with ubiquinol will additionally be compared with placebo, and ubiquinol will be compared with ubiquinol + D-ribose). However, our sample sizes will be able to detect effect sizes of at least 0.7 in absolute value with at least 90% power. Analogous computations and tests will be made to examine the effects of ubiquinol, D-ribose, and the combination of ubiquinol with D-ribose on PAP changes. The large and clearly clinically important effect size of ubiquinol on PAP observed in our pilot study (− 2.8) suggests that our sample sizes will provide 99% power for at least the ubiquinol-placebo comparison.

For hypothesis #4, a two-tailed independent samples *t*-test will be used to test the null hypothesis that the mean change in the 6MWT from baseline to the end of 12 weeks in patients taking ubiquinol is different from the mean change for placebo. Comparisons of D-ribose versus placebo, of ubiquinol + D-ribose versus placebo, and of ubiquinol versus ubiquinol + D-ribose will likewise be made. Our proposed sample sizes (62 per group) will allow detecting Cohen effect sizes on the 6MWT of at least 0.7 with at least 90% power at a nominal 0.05 level of significance with Bonferroni correction for four comparisons.

For hypothesis #5, our pilot data suggested that ubiquinol has a large effect size on BNP (− 1.5). Thus, two-tailed independent samples t-tests comparing changes in BNP from baseline to the end of 12 weeks will have a large power (99%) for investigating Hypothesis #5 using Bonferroni corrections. In general, our sample sizes will be able to detect effect sizes of at least 0.7 in absolute value with at least 90% power.

Finally, hypothesis #6, the change in lactate/ATP ratio from baseline to 12 weeks will be computed for each patient. A one-tailed independent samples *t*-test will examine the null hypothesis of no differences in mean changes between ubiquinol and placebo groups against the alternative that the mean change in patients on ubiquinol will be numerically smaller, which includes the possibility of a negative change. Similar tests will compare D-ribose versus placebo, the combination of ubiquinol + D-ribose versus placebo, and ubiquinol versus ubiquinol + D-ribose. Our sample sizes will give us a power of at least 90% to detect effect sizes of at least 0.7 in absolute value by using a 0.05 significance level and Bonferroni correction for the four comparisons.

We will also examine whether there is an interactive effect with synergism on KCCQ scores when simultaneously administering ubiquinol and D-ribose. To investigate whether ubiquinol and D-ribose interact synergistically, a linear regression model of KCCQ score change from baseline to week 12 will be built. The model will include indicators for ubiquinol and D-ribose treatment as independent variables, as well as the product of these two indicators (the interaction term), gender, and race/ethnicity demographics. Analogous regressions will be conducted with KCCQ subscales, with the POMS Vigor score, as well as with indicators of left ventricular diastolic function. In addition, this study will allow us to: (1) examine the mediating role of the lactate/ATP ratio in the effect of ubiquinol on left ventricular diastolic function in patients; and (2) examine whether there is an interactive effect with synergism on this function when simultaneously administering ubiquinol and D-ribose. A structural equation model will be built to explore our mechanistic hypothesis that a reduction in lactate/ATP ratio mediates the association between ubiquinol treatment and reduction in E/e’ ratio. We will control for potential confounders such as demographics, baseline lactate/ATP ratio, and baseline E/e’ ratio. This modeling approach will take full advantage of the fact that subjects will provide measures of lactate/ATP and E/e’ ratios at two time points (baseline and 12 weeks), which will allow disentangling the dynamic effects of time, a crucial variable in causality analyses. Similar models will be built by using the other indicators of left ventricular diastolic function in place of E/e’. To investigate whether ubiquinol and D-ribose interact synergistically, a linear regression model of E/e’ change from baseline to week 12 will be built that will also include as independent variables indicators for ubiquinol and D-ribose treatment, as well as the product of these two indicators (the interaction term), gender, and race/ethnicity. Analogous regressions will be conducted with other indicators of left ventricular diastolic function. If necessary, transformations will be performed to achieve the assumptions of regression models.

To reduce missing data during study documentation, research personnel will be trained to watch for missing data on forms and within the data software program. There will be regular monitoring of missing data and enhanced subject contact by phone. The study statisticians will use analytic techniques to estimate causal effects in the presence of missing data such as multiple imputation and likelihood-based analysis.

### Adverse events

Adverse occurrences or unanticipated problems reported by a subject during the course of their participation in the study will be immediately documented according to study protocol. A report will be sent to IRB and the data and safety monitoring board (DSMB) on the appropriate forms. For each occurrence, the DSMB will evaluate independently to determine attribution of the occurrence to the study protocol or not and report to National Institute of Aging (NIA) per policy. All DSMB members are available in real time to review and recommend appropriate actions regarding any adverse or unanticipated events or other safety issues. Any action taken to suspend or terminate the project will be reported immediately to KUMC IRB, NIH Office of Sponsored Projects, and the study Program Officer at NIA.

### Protection against risks

Although no appreciable risk of physical or mental harm is expected to result from the proposed protocol, procedures for dealing with adverse effects are established. Participants will be instructed to take the supplements with a meal to prevent or minimize gastrointestinal symptoms. The procedures listed below will be followed to avert negative reactions to procedures or questions posed during the CTSU visits or the follow-up telephone calls: 1) all subjects will be informed that they are free to skip questions, 2) end data collection, 3) not participate in any component of clinical evaluation and laboratory testing, or 4) withdraw from the study without changes in their standard or usual medical or nursing care. Subjects will be instructed to report immediately to the PI, Project Director, Research Associate or the cardiologists any negative side effects when consuming the supplements. Appointments will be cancelled and rescheduled if subjects express an inability to travel for scheduled CTSU visits. Furthermore, personnel are trained to discontinue any data collection in which the participant becomes emotionally upset or notes discomfort. The Research Associate or the PI will refer that person to our cardiologists or call 911, if immediate medical assistance is required. Subjects who express worry or distress from perceived loss of privacy, have concerns about inconvenience or rights as a research subject, will be assured that they do not have to participate, that their medical care will not be affected, or if already enrolled in the study that they may withdraw at any time without prejudice. In addition, any such concerns will be documented and reported to IRB. If participants experience serious adverse effects deemed by the investigators to be caused by the investigational supplements, the participant will be notified in person or via phone by the investigators, Program Director, or Research Associate to discontinue usage immediately.

### Interim analysis

There are no advance plans for interim and/or futility analysis at this time.

### Data and safety monitoring

The PI will be responsible for ensuring participants’ safety on a daily basis. The DSMB appointed by the NIA, will act in an advisory capacity to the NIA Director to monitor participant safety, evaluate the progress of the study, to review procedures for maintaining the confidentiality of data, the quality of data collection, management, and analyses. The content of the data and safety monitoring report will include: study status, participant descriptive information, safety information, and study quality. Prior to subject recruitment, the members of the DSMB will read the written study protocols, informed consent procedures, data safety monitoring policies, and plan. Members of the DSMB will initially discuss the plan for clarification of their responsibilities, the board structure, and organization and rules of operation, as noted in the policies. The DSMB appointed committee will adhere to the DSMB Charter outlined by NIA. All trial progress and DSMB advice on continuation, modification, or termination are reported to the PI, IRB, and the NIA Program Officer. In addition, the University has a Human Research Protection Program in place in which random audits are conducted for quality assurance.

### Dissemination policy

The researchers plan to disseminate the trial results by oral presentations, publications, registry (ClinicalTrial.gov.), and other NIH reports to promote broad and responsible dissemination of the data. The authors will include names of study researchers and individuals who significantly contribution to the conceptualization, design, execution, or interpretation of the research, as well as to the drafting or substantively reviewing or revising the manuscripts. Since this is a NIH funded trial, it is registered on ClinicalTrials.gov and the results will be submitted to ClinicalTrials.gov. In addition, the investigators will submit final peer-reviewed journal manuscripts that arise from NIH funds to PubMed Central immediately upon acceptance for publication.

## Discussion

The prevalence of HF is increasing, and it is estimated that there will be 8.0 million U.S. adults who have HF by 2030 [43]. More than 50% of HF patients have a type of HFpEF, also termed diastolic HF [44]. The hallmark symptoms of HFpEF are lack of energy, fatigue, shortness of breath, exercise intolerance, and decreased quality of life [45]. Thus, patients with HFpEF suffer with severe lack of energy and other debilitating symptoms that affect their overall health-related quality of life. We have pilot data on patients with HFpEF showing that two supplements (ubiquinol and D-ribose) added to their usual HF care enhanced their myocardial energetics and diastolic function resulting in decreased symptom burden. Both ubiquinol (active form of coenzyme Q10) and D-ribose play a vital role in mitochondrial ATP production.

This study will provide us an opportunity to translate what we have observed in the laboratory and in the cardiovascular clinic to decrease patients’ debilitating symptoms of HFpEF. In a rat model, we have observed a significant decrease in myocardial nuclear damage (apoptosis) and mitochondrial superoxide production associated with ubiquinol administration during an acute oxidative stress condition. Mitochondrial damage as assessed by electron microscopy was greatly reduced with the administration of ubiquinol. In the clinic setting, we have observed significant improvements in cardiac function leading to improved symptoms in patients with HFpEF who received ubiquinol and/or D-ribose. Translating these significant findings via a human study will provide essential data to support the use of these supplements in clinical practice.

With the new advances in bioenergetics, clinicians can better understand HFpEF and the debilitating symptoms that accompany this illness. We are proposing research to develop improved, personalized strategies to treat the adverse symptoms of chronic HFpEF by comparing the effectiveness of usual care with alternative treatments. By measuring both biological and behavioral dynamics of symptoms, the sentinel data from this proposed study could change the trajectory of HFpEF.

## Abbreviations

6MWT: 6-min walk test
AA: African Americans
ACC: American College of Cardiology
AHA: American Heart Association
AMP: Adenosine monophosphate
ATP: Adenosine triphosphate
BNP: B-type natriuretic peptide
CTSU: Clinical and Translational Science Unit
DSMB: Data and safety monitoring board
EF: Ejection fraction
ETC: Electron transport chain
HF: Heart failure
HFpEF: Heart failure with preserved left ventricular ejection fraction
IRB: Institutional Review Board
KCCQ: Kansas City Cardiomyopathy Questionnaire
LVEF: Left ventricular ejection fraction
NIA: National Institute of Aging
NIH: National Institutes of Health
PI: Principal Investigator
POMS: Profile of Mood States
